# *Tg*CDPK3 Regulates Calcium-Dependent Egress of *Toxoplasma gondii* from Host Cells

**DOI:** 10.1371/journal.ppat.1003066

**Published:** 2012-12-04

**Authors:** James M. McCoy, Lachlan Whitehead, Giel G. van Dooren, Christopher J. Tonkin

**Affiliations:** 1 The Walter and Eliza Hall Institute of Medical Research, Melbourne, Australia; 2 Department of Medical Biology, University of Melbourne, Melbourne, Australia; 3 Research School of Biology, Australian National University, Canberra, Australian Capital Territory, Australia; University of Geneva, Switzerland

## Abstract

The phylum Apicomplexa comprises a group of obligate intracellular parasites of broad medical and agricultural significance, including *Toxoplasma gondii* and the malaria-causing *Plasmodium* spp. Key to their parasitic lifestyle is the need to egress from an infected cell, actively move through tissue, and reinvade another cell, thus perpetuating infection. Ca^2+^-mediated signaling events modulate key steps required for host cell egress, invasion and motility, including secretion of microneme organelles and activation of the force-generating actomyosin-based motor. Here we show that a plant-like Calcium-Dependent Protein Kinase (CDPK) in *T. gondii*, *Tg*CDPK3, which localizes to the inner side of the plasma membrane, is not essential to the parasite but is required for optimal *in vitro* growth. We demonstrate that *Tg*CDPK3, the orthologue of *Plasmodium Pf*CDPK1, regulates Ca^2+^ ionophore- and DTT-induced host cell egress, but not motility or invasion. Furthermore, we show that targeting to the inner side of the plasma membrane by dual acylation is required for its activity. Interestingly, *Tg*CDPK3 regulates microneme secretion when parasites are intracellular but not extracellular. Indeed, the requirement for *Tg*CDPK3 is most likely determined by the high K^+^ concentration of the host cell. Our results therefore suggest that *Tg*CDPK3's role differs from that previously hypothesized, and rather support a model where this kinase plays a role in rapidly responding to Ca^2+^ signaling in specific ionic environments to upregulate multiple processes required for gliding motility.

## Author Summary

The phylum Apicomplexa includes a number of medically and agriculturally relevant parasites. These include the *Plasmodium* species, agents of malaria and estimated to cause over 1 million deaths per year, and *Toxoplasma gondii*, which infects 30–80% of any human population. These parasites rely on a unique form of actomyosin-powered motility to perpetuate infection, but the molecular mechanisms regulating this vital process are virtually unknown. Here, we describe a plant-like Ca^2+^-dependent kinase of *T. gondii*, *Tg*CDPK3, which is involved in the rapid activation of egress from host cells during Ca^2+^ signaling. *T. gondii*'s requirement for *Tg*CDPK3 seems to rely specifically on the local ionic environment, being dispensable in conditions typical of the extracellular environment. Activity is also dependent on localization to the parasite plasma membrane, which appears to be conferred by a consensus motif at the kinase N-terminus that is typically acylated. This work provides some of the first insights into the intricacies of signaling pathways regulating apicomplexan motility.

## Introduction

*Toxoplasma gondii*, the causative agent of toxoplasmosis, is a member of the phylum Apicomplexa, a group of unicellular eukaryotes consisting chiefly of obligate intracellular parasites. Among these medically and agriculturally important parasites are the *Eimeria* spp., causing enteritis in poultry and cattle; the *Cryptosporidium* spp., opportunistic agents of diarrhea; and the agents of malaria, the *Plasmodium* spp. *T. gondii* is the most ubiquitous of the Apicomplexa, being able to infect virtually any nucleated cell from a range of mammalian and avian species. *T. gondii* infects 30–80% of any human population, though on initial contact typically causes only mild or asymptomatic infections [1]. However, infection during pregnancy, or reactivation of latent cysts in immunocompromised patients, can cause severe disease. Furthermore, loci of severe disease in immunocompetent patients are uncommon but significant in their effect [2], [3], [4]. For example, chronic infection of retinal tissue is thought to be a cause of high levels of blindness in some countries [5].

Despite the diversity of cell types and hosts targeted, the Apicomplexa show significant conservation in the mechanisms used to move through tissue and invade host cells, and the structures vital to these processes. The phylum's namesake, the apical complex, defines the apical tip of parasites, and comprises a microtubule-organizing centre [6] and the rhoptry and microneme organelles [7]. Secretion of the contents of these apical organelles is tightly coordinated with activity of an unique actomyosin motor, known as the glideosome [8], [9], [10], [11], [12], [13], [14]. Housed in the pellicular space between the parasite plasma membrane and a network of flattened cisternae underlying it, known as the inner membrane complex (IMC), activity of the glideosome drives parasite motility during host cell egress, tissue traversal, and host cell invasion. Seen in terms of the *T. gondii* asexual lifecycle, the standard model of motility states that following intracellular replication parasites activate secretion of the micronemes. These contain a perforin-like protein (*Tg*PLP1), required for lysis of the parasitophorous vacuole membrane (PVM), as well as adhesins that are secreted onto the parasite surface and bind extracellular receptors [7], [15]. These transmembrane adhesins then link, through short cytoplasmic tails, to the glideosome. Coincident activation of the actomyosin motor drags the adhesins rearwards across the surface of the parasite, in turn pulling it forward. Parasites thereby escape the host cell, and “glide” through tissue. The asexual lifecycle is then completed upon recognition and invasion of a new host cell, where the same processes that allowed host cell escape now allow invasion. The current model of the asexual lifecycle describes these three motile stages of host cell egress, motility, and invasion as different expressions of a single, active, parasite-driven process: gliding motility [16].

Although work with *T. gondii* has provided significant insight into the mechanics of apicomplexan gliding motility, very little is known about how it is regulated. Early studies suggested that calcium signaling pathways play a crucial role, as calcium ionophores can be used to stimulate microneme secretion and glideosome activity, whereas calcium chelators inhibit this [17], [18]. In activating gliding motility through these pathways *T. gondii* appears to sense and respond to its environment, releasing calcium from intracellular stores by a variety of means whose mechanics have been only hinted at [19]. Accumulation of abscisic acid by replicating parasites as a quorum sensing-like system, and detection of a local reducing environment by NTPases in the parasitophorous vacuole, both stimulate calcium-dependent egress from host cells in *T. gondii* [20], [21]. In *P. falciparum*, calcium-stimulated secretion and engagement of micronemal adhesins with red blood cell receptors during host cell invasion causes sequential release of rhoptry contents and dampening of the calcium signal [22]. However the best characterized of these environmental cues is the local environmental potassium concentration. Sensing extracellular potassium has been suggested to enable *T. gondii* and *P. falciparum* to determine extracellularity, and is important in regulating parasite cytoplasmic calcium levels and activating motility [22], [23]. But beyond these insights, which rely heavily on the use of pharmacological agents, the molecular mechanisms underlying calcium-mediated signal transduction pathways during gliding motility remain largely elusive.

In other systems intracellular calcium flux is commonly translated into cellular responses by activation of protein kinases. As such, a group of plant-like Calcium-Dependent Protein Kinases (CDPKs) has received significant attention as potential hubs in apicomplexan signal transduction cascades. The CDPKs belong to a superfamily of kinases prominent in the calcium signaling cascades of plants and some ciliates but absent from the genomes of animals and fungi. They are therefore touted as potential drug targets [24], [25]. The domain structure of these kinases consists of a variable N-terminal region, which is involved in substrate recognition and protein interaction [26], [27], a kinase catalytic domain, and a regulatory domain which itself consists of an autoinhibitory junction domain and a calmodulin-like domain (CLD) [28], [29]. The CLD is comprised of four EF hands that, upon binding calcium, effect a dramatic structural change that extricates the junction domain from its autoinhibitory interaction with the substrate-binding site of the kinase domain. This activates kinase domain catalytic activity [28], [30].

Recently, apicomplexan CDPKs have been implicated as key effectors of calcium signal transduction cascades in a number of processes [25]. For example, conditional expression systems and small molecule inhibitor studies of *T. gondii* CDPK1 (*Tg*CDPK1) have demonstrated its importance in regulating microneme secretion [31], [32], [33]. In the *Plasmodium* species, *P. falciparum* PfCDPK5 regulates parasite egress from host cells [34], *P. berghei Pb*CDPK3 is required for ookinete traversal of the mosquito midgut epithelium [35], while *Pb*CDPK4 is involved in development of the male gametocyte [36].

CDPKs have also been suggested to be regulators of the glideosome. Glideosome proteins are heavily phosphorylated, often in a calcium-dependent manner [37]. In this regard, *P. falciparum* CDPK1 (*Pf*CDPK1) has been of significant interest because it has been demonstrated to be co-expressed with genes coding for glideosome component proteins [38], and can phosphorylate glideosome components *in vitro* [39]. *Pf*CDPK1 co-localizes with the glideosome at the parasite periphery in schizonts and merozoites, and treatment with small molecular inhibitors of *Pf*CDPK1 has been shown to cause a block in late-stage schizont development [38]. Despite this work, the physiological targets and *in vivo* role of *Pf*CDPK1 are yet to be determined. Furthermore an argument for a role in glideosome phosphorylation has been complicated by the recent revelation that *P. berghei* CDPK1 in fact regulates transcription of stored mRNA during ookinete development in the mosquito midgut [40].

In the present study we show that the orthologue of *Pf*CDPK1 in *T. gondii*, *Tg*CDPK3, co-localizes with the glideosome by targeting to the parasite plasma membrane through a dual acylation motif similar to that of *Pf*CDPK1. This membrane localization appears to be vital for *Tg*CDPK3's activity, suggesting its substrates are also membrane-embedded. We then show that *Tg*CDPK3 is dispensable for completion of the lytic cycle, though its knockout does slow *in vitro* growth. This deficiency appears to be due to a specific role for *Tg*CDPK3 in upregulating calcium-dependent processes required for gliding motility during host cell egress. Upon calcium ionophore stimulation of egress, *Tg*CDPK3-deficient parasites were unable to permeabilize the PVM, were deficient in microneme secretion, and could not activate gliding motility. However, extracellular parasites showed no defects in motility, invasion or microneme secretion following host cell escape. Indeed, we demonstrate that *Tg*CDPK3 is only required for calcium-mediated signaling events in a high potassium environment typical of the host cell. Our data indicate that, contrary to hypotheses for a role in direct glideosome phosphorylation, *Tg*CDPK3 likely acts in the calcium signaling transduction cascade upstream of a number of egress-required events.

## Results

### Dual acylation of TgCDPK3 is necessary and sufficient for localization to the parasite membrane

**Figure 1:**
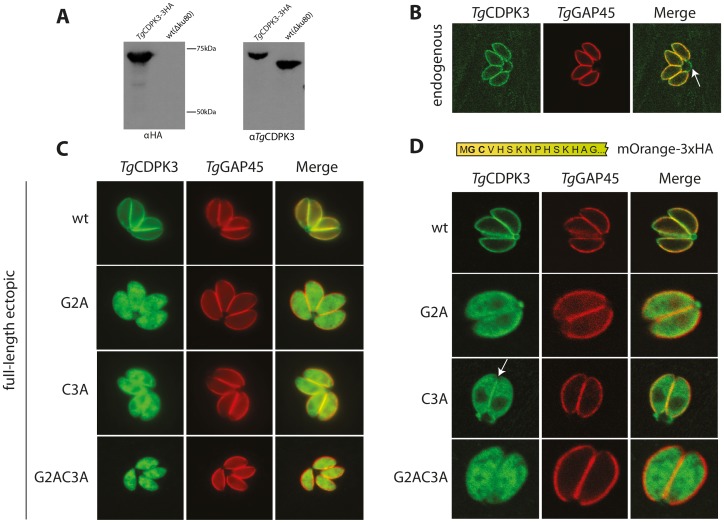
*Tg*CDPK3 localizes to the plasma membrane through a putative N-terminal acylation motif. A) Tagging of the *tgcpkd3* endogenous locus with 3HA. *Tg*CDPK3-3HA runs at the expected size by western blot, showing a size shift corresponding to the addition of the 3HA epitope by probing with α*Tg*CDPK3. *Tg*CDPK3-3HA shows clear banding using αHA, with no band seen in wild-type (wt(ΔKu80)) parasites. B) Staining *Tg*CDPK3-3HA parasites with αHA and α*Tg*GAP45 shows co-localization between *Tg*CDPK3 and *Tg*GAP45 at the parasite periphery, likely through plasma membrane targeting as judged by staining of the parasite residual body (white arrow). C) Substitution mutations of the putative acylated residues Gly2 and Cys3 in full-length ectopic copies of *Tg*CDPK3 disrupts its peripheral targeting, showing these residues are necessary for its localization. D) The 15 most N-terminal amino acids of *Tg*CDPK3 impart plasma membrane localization to mOrange fluorescent protein, but mutations in the putative acylated residues identical to those described above disrupt this pattern. White arrow = concentration of *Tg*CDPK3_NC3A_ at the parasite periphery.

**Figure 2:**
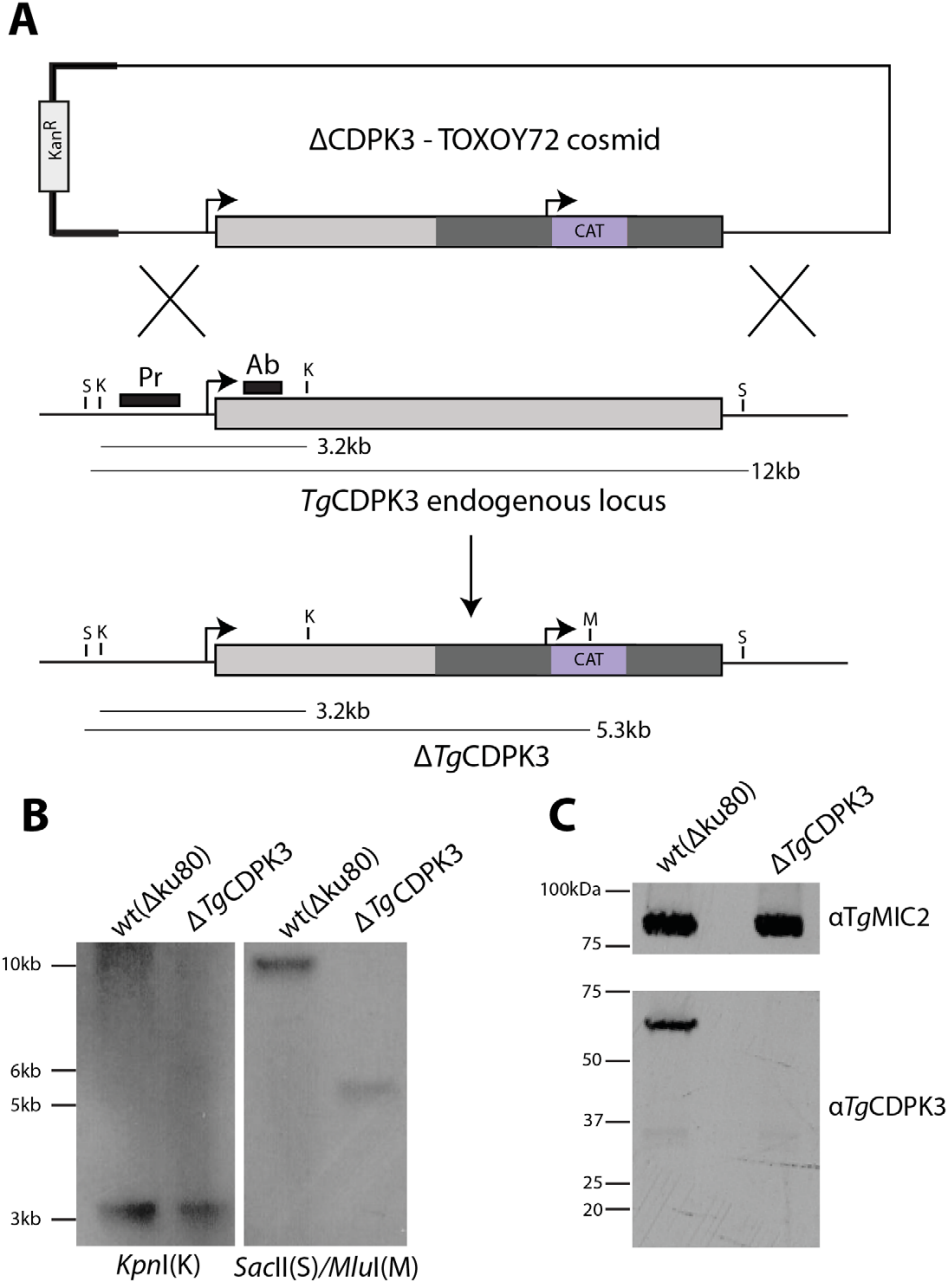
Knockout of *Tg*CDPK3 by double homologous recombination. Wild-type (ΔKu80) = wild-type parasites. A) Design of ΔCDPK3-TOXOY72 cosmid construct, whereby double homologous recombination replaces the 3′ half of the genomic locus of *Tg*CDPK3 with a CAT resistance cassette. Bold line shows TOXOY72 cosmid backbone. Schematic also shows hybridization site for Southern blot probe (“Pr”), recognition site for α*Tg*CDPK3 antibody (“Ab”), and restriction sites used in Southern blotting (K = *Kpn*I, M = *Mlu*I, S = *Sac*II). B) Southern blot analyses of Δ*Tg*CDPK3. *Kpn*I digest control shows identical banding pattern between wild-type and Δ*Tg*CDPK3 parasites, whereas digestion of gDNA with *Sac*II/*Mlu*I shows the expected size drop of in banding between wild-type and Δ*Tg*CDPK3 (“*”). C) Knockout of a clonal transgenic line, Δ*Tg*CDPK3, was confirmed with α*Tg*CDPK3 antibody, showing lack of any significant banding by western blot compared to wild-type parasites. α*Tg*MIC2 loading control shows equal loading of parasite lysates.

To start our analysis of *Tg*CDPK3 we wished to determine its subcellular localization. To do this we tagged the C-terminus of the endogenous locus of *Tg*CDPK3 with a triple HA tag (3HA), using the RH Δ*ku80* (ΔKu80) parasite line. Tagging in the resulting parasite line, *Tg*CDPK3-3HA, was confirmed by western blot, noting a strong signal upon probing with αHA antibodies (Fig. 1A). Probing of the same lysate with an α-*Tg*CDPK3 antibody (Fig. 2A), created by immunization with a peptide consisting of a 14 amino acid stretch from the N-terminal variable region of *Tg*CDPK3, showed a size shift in *Tg*CDPK3-3HA compared to ΔKu80, corresponding to the size of the 3HA tag (Fig. 1A). *Tg*CDPK3-3HA was observed to co-localize with *Tg*GAP45 of the glideosome at the parasite periphery by IFA (Fig. 1B). Interestingly, *Tg*CDPK3-3HA clearly targeted to the residual body between replicating parasites (Fig. 1B - white arrow), suggesting this kinase is in fact anchored to the plasma membrane in *T. gondii*.

A number of plant CDPKs are targeted to cell membranes by dual acylation of an N-terminal consensus motif [24], [41]. This motif is also found in apicomplexan CDPKs, including *Tg*CDPK3's orthologue, *Pf*CDPK1 [42]. In this motif a glycine residue at amino acid position 2 is typically myristoylated, which then acts as a recognition site for palmitoylation of a nearby cysteine [43]. In analyzing the N-terminal sequence of *Tg*CDPK3 we noted it contains a glycine residue at position 2 (Gly2) followed by a cysteine at position 3 (Cys3). To test whether these residues might be acylated in targeting of *Tg*CDPK3 to the plasma membrane, vectors were constructed containing a 3HA-tagged cDNA (ectopic) copy of the wild-type *Tg*CDPK3 coding sequence (*Tg*CDPK3_wt_), or copies containing alanine substitution mutations in the putative N-terminal acylation motif. These vectors were integrated into the non-essential UPRT locus in ΔKu80 parasites, resulting in lines containing either wild-type *Tg*CDPK3 (*Tg*CDPK3_wt_), a single mutation in the myristoylation site (*Tg*CDPK3_G2A_), a single palmitoylation site mutant (*Tg*CDPK3_C3A_), or a double mutant (*Tg*CDPK3_G2AC3A_), wherein both myristoylation and palmitoylation are predicted to be perturbed. While *Tg*CDPK3_wt_ showed localization identical to endogenous *Tg*CDPK3-3HA, mutation in Gly2 or Cys3, or both, disrupted membrane targeting and caused an essentially cytoplasmic distribution pattern (Fig. 1C). This suggests that the putative N-terminal acylation motif of *Tg*CDPK3 is necessary for its plasma membrane localization.

We wanted to also know if this motif was sufficient for *Tg*CDPK3's localization. To this end, a cDNA sequence encoding the 15 most N-terminal residues of *Tg*CDPK3, coding either the wild-type sequence or substitution mutations identical to those described above, was fused to mOrange and 3HA tags (Fig. 1D). Transfection into RH *Δhxgprt* (ΔHx) parasites resulted in the lines *Tg*CDPK3_Nwt_, *Tg*CDPK3_NG2A_, *Tg*CDPK3_NC3A_, and *Tg*CDPK3_NG2AC3A_. The wild-type N-terminal sequence showed clear plasma membrane localization indistinguishable from that of *Tg*CDPK3-3HA (Fig. 1D). Mutations in the putative acylation sites again resulted in cytoplasmic distribution of *Tg*CDPK3_NG2A_, *Tg*CDPK3_NC3A_ and *Tg*CDPK3_NG2AC3A_ similar to that seen for the full-length N-terminal mutant proteins. It is also noteworthy that a small concentration of *Tg*CDPK3_NC3A_ could be observed at the periphery of parasites, suggesting an amount of membrane targeting was possible if the putative glycine myristoylation site was retained (Fig. 1D - white arrow). Together, these data demonstrate that the N-terminal Gly2 and Cys3 residues of a putative *Tg*CDPK3 membrane-targeting motif are both necessary and sufficient for high affinity localization to the plasma membrane, likely through modification by acylation.

### *Tg*CDPK3 plays an important role in the calcium-dependent egress of parasites from host cells, but not in motility or host cell invasion

To understand the role of *Tg*CDPK3 in *T. gondii* we disrupted the endogenous genetic locus by double homologous recombination. To ensure high efficiency of recombination we made use of cosmid recombineering to create the knockout construct, in conjunction with the ΔKu80 strain [44], [45]. The end-sequenced cosmid TOXOY72 was identified through the *toxodb.org* genome browser to cover the genomic locus of *tgcdpk3* [46]. TOXOY72 was modified to replace a region of *tgcdpk3* (containing subdomains X-XII of the kinase domain, through the remaining 3′ half of its coding sequence) with the chloramphenicol acetyl transferase (CAT) selectable marker (Fig. 2A).

Following transfection into ΔKu80, a stable transgenic line was selected on chloramphenicol. Clonal lines were then screened for successful disruption by PCR (data not shown). Southern blot of candidate clones was fully consistent with the expected knockout locus (Fig. 2B).

To confirm *Tg*CDPK3-deficiency in transgenic parasites, we analyzed its expression in our knockout line. Using anti-TgCDPK3 antibodies, no *Tg*CDPK3 expression could then be seen in the knockout (Fig. 2C). Additionally no product of a lower molecular weight was seen, suggesting that successful disruption and truncation of the 3′ half of *tgcpdk3* caused instability of the resulting mRNA transcript or protein, rendering this parasite a complete knockout (Δ*Tg*CDPK3).

**Figure 3:**
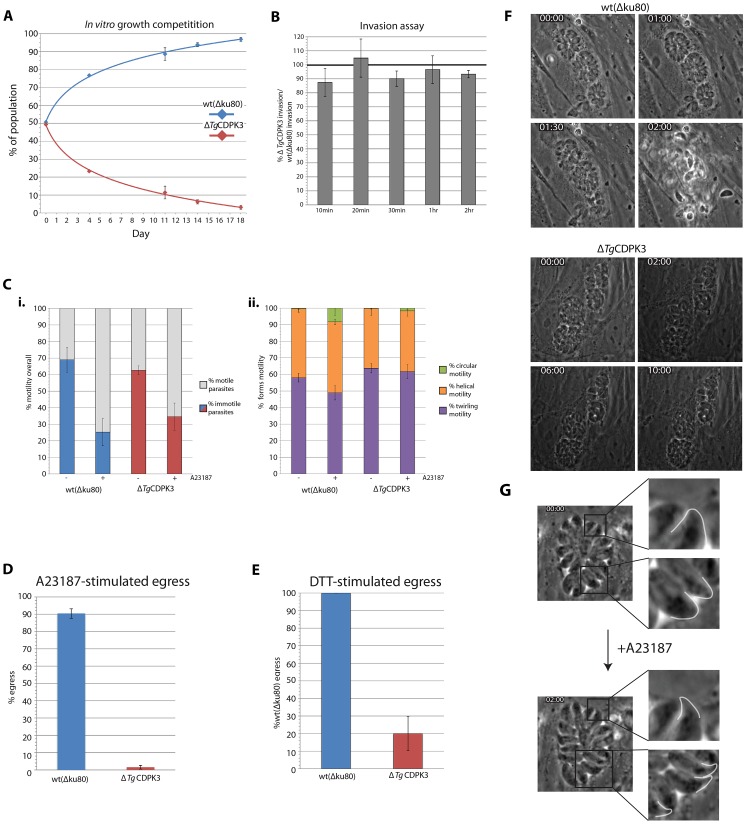
*Tg*CDPK3 is required for calcium ionophore-induced egress from host cells. A) Mixing wild-type and Δ*Tg*CDPK3 parasites shows knockout of *Tg*CDPK3 causes *T. gondii* to be inefficient in ability to complete the lytic lifecycle *in vitro*, being effectively out-competed by wild-type by day 18 of the experiment. B) Invasion rates of wild-type and Δ*Tg*CDPK3 parasites shows no significant difference over a range of time points. C) Live motility assay of wild-type and Δ*Tg*CDPK3 parasites. i. Overall proportion of motile versus immotile parasites between wild-type and Δ*Tg*CDPK3 parasites, either with or without calcium ionophore A23187 stimulation. Both strains show amplification of motility following A23187 treatment, with no significant difference in level of amplification. ii. Proportions of twirling, helical and circular motility exhibited by wild-type and Δ*Tg*CDPK3 parasites. Δ*Tg*CDPK3 parasites show a slight preference for twirling motility over helical following A23187 stimulation, as compared to wild-type. D) Δ*Tg*CDPK3 mutants show a severe defect in ability to egress from host cells following stimulation of calcium signaling with calcium ionophore A23187. E) Δ*Tg*CDPK3 mutants show a defect in egress from host cells following stimulation of calcium signaling with reducing agent DTT. Egress levels are normalized against wild-type. F) Live time-lapse microscopy of Δ*Tg*CDPK3 parasites show an inability to activate motility and escape host cells up to and beyond 10 min after ionophore stimulation, whereas wild-type parasites activate egress within 1.30 min. Calcium ionophore is added at 30 sec time point. G) An inability to activate egress upon ionophore stimulation is not due to a general defect in calcium signaling in Δ*Tg*CDPK3, as mutants show extrusion of conoids coincident with the normal timing of wild-type extrusion and egress.

To investigate *Tg*CDPK3's function we first analyzed the effect of knockout of *Tg*CDPK3 on efficient completion of the asexual lytic lifecycle. To monitor this sensitively we performed a competition assay, mixing equal amounts of freshly lysed Δ*Tg*CDPK3 and wild-type parasites and adding ∼2.5×10^5^ mixed parasites to culture. Upon complete lysis of HFF monolayers, 2.5×10^5^ parasites were passed to fresh HFFs, and at each passage the proportion of Δ*Tg*CDPK3 parasites in the population was monitored by IFA by staining for the CAT selectable marker. Here, we saw that Δ*Tg*CDPK3 mutants have a lytic cycle defect, resulting in them being effectively outcompeted by wild-type parasites by day 18 of the experiment (Fig. 3A).

To analyze where in the lytic cycle this defect lay we monitored the ability of Δ*Tg*CDPK3 to perform motility, invasion and host cell egress – all processes requiring active gliding motility stimulated through calcium signaling. We began by assaying the invasion capacity of Δ*Tg*CDPK3 mutants, using the two-color invasion assay previously described [14]. Parasites were allowed to invade host cells over a range of time points (from 10 min to 2 hr) in order to capture any difference in invasion rate. We saw no consistent, significant difference in invasion capacity of Δ*Tg*CDPK3 parasites compared to wild-type, suggesting that *Tg*CDPK3 has no direct role in gliding motility during host cell invasion of *T. gondii* (Fig. 3B).

We next assayed motility of Δ*Tg*CDPK3 parasites. Wild-type and Δ*Tg*CDPK3 parasites were settled onto poly-L-lysine coated plates and motility was recorded by time-lapse microscopy. Looking at overall levels of motility, we saw no significant difference between wild-type and *ΔTg*CDPK3 parasites (Fig. 3Ci). Motility of parasites can be enhanced by stimulation with a calcium ionophore, such as A23187. Wild-type and Δ*Tg*CDPK3 parasites amplified motility to similar levels following ionophore treatment. We then assayed for specific perturbations in the different types of motility performed by *T. gondii*; twirling, helical, or circular [47]. We noted little difference between Δ*Tg*CDPK3 and wild-type (Fig. 3Cii). However, it is interesting to note that A23187 treatment slightly increased the amount of circular motility of wild-type parasites, which was not so pronounced in Δ*Tg*CDPK3 parasites. This however, remains statistically insignificant (Fig. 3Cii).

We then investigated the ability of Δ*Tg*CDPK3 parasites to perform calcium ionophore-induced host cell egress. Calcium-dependent gliding motility in late stage (∼30 hr growth) Δ*Tg*CDPK3 and wild-type parasite vacuoles was stimulated with 8 µM calcium ionophore A23187 for 3 min, and the percentage of egressed vacuoles determined by IFA. Using this assay, Δ*Tg*CDPK3 mutants have an almost complete ablation of egress (1.5% of vacuoles showed egress, as compared to 90% ΔKu80), highlighting a role for *Tg*CDPK3 in this process (Fig. 3D). We also stimulated calcium-dependent egress by treatment with the reducing agent dithiothreotol (DTT) [21]. Δ*Tg*CDPK3 and wild-type parasites were treated with 5 mM DTT for 15 min and the percentage egress was then calculated, normalizing Δ*Tg*CDPK3 egress rates to wild-type. Again, Δ*Tg*CDPK3 parasites showed significantly lower levels of egress (Fig. 3E). This suggests *Tg*CDPK3 is important in general for response to egress stimuli, although higher relative levels of egress were noted than following A23187 stimulation.

To better understand the role of *Tg*CDPK3 in host cell egress we imaged parasites live using time-lapse microscopy. Wild-type parasites were seen to activate gliding motility and escape host cells 1–1.30 min following stimulation with 8 µM A23187 (Fig. 3F, Video S1). Δ*Tg*CDPK3 mutants, on the other hand, were generally immotile and did not activate gliding motility, remaining intracellular for 10 min or more following ionophore addition (Fig. 3F, Video S2). Occasionally, parasites did activate delayed gliding motility ∼5–10 min after ionophore addition. If this was the case, mutants were able to glide and reinvade fresh host cells normally (data not shown). Importantly, however, Δ*Tg*CDPK3 parasites were able to extrude their conoid (which is thought to be a calcium-dependent event) following ionophore stimulation (Fig. 3G, Video S3). In live imaging, wild-type parasites extended the conoid immediately prior to egress (∼1–1.30 min following A23187 stimulation) (data not shown). Timing of conoid extrusion in Δ*Tg*CDPK3 mutants was identical to this. This is indicative of Δ*Tg*CDPK3 mutants having a specific, rather than general, block in responding to calcium flux upon stimulation.

### *Tg*CDPK3 controls the calcium-dependent permeabilization of the PVM and intracellular microneme release

Together, the above data suggested that *Tg*CDPK3 is not generally required for *T. gondii* gliding motility. Instead, this kinase appears to be involved in the specific upregulation of calcium-dependent processes required during host cell egress. We were interested in what way *Tg*CDPK3 might act in these egress-specific pathways. We noted during live time-lapse imaging that the PVM appeared intact in Δ*Tg*CDPK3 vacuoles, as parasites slightly rearranged and extended conoids only within a limited space (Videos S2, S3). This is in contrast to other glideosome mutants that are unable to activate motility but can break down the PVM during activation of egress [48]. PVM permeabilization does not require gliding motility but rather calcium-dependent secretion of the perforin-like protein *Tg*PLP1 from the micronemes [15].

**Figure 4:**
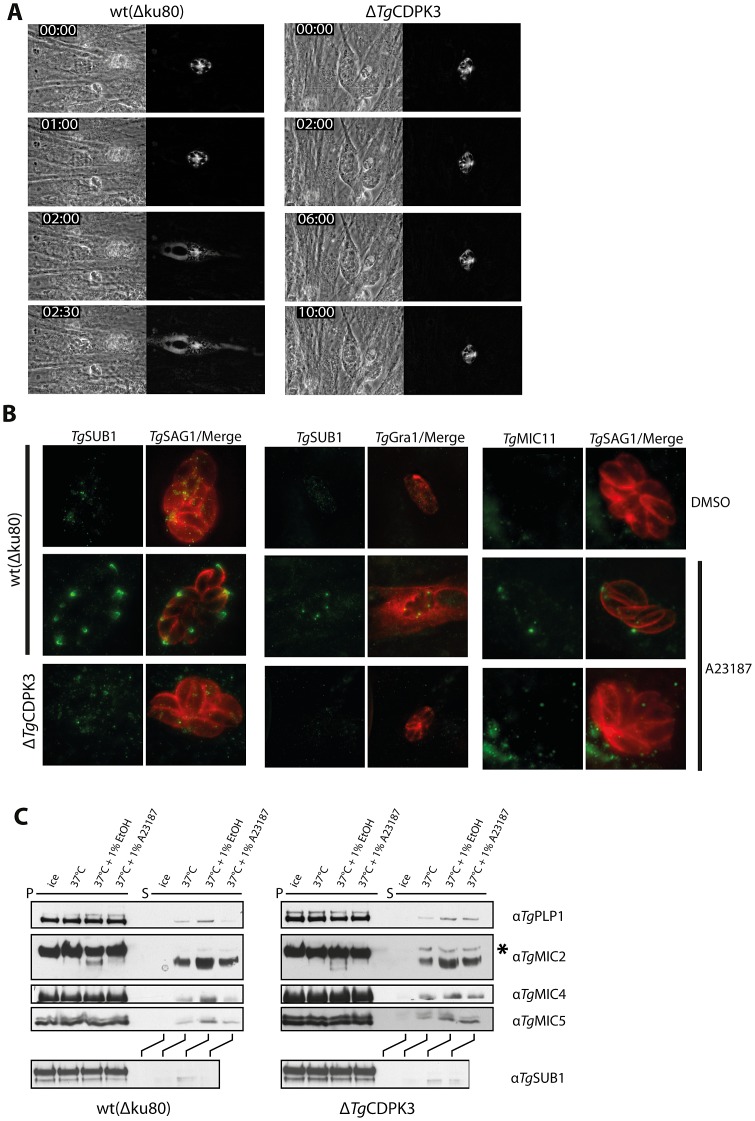
Intracellular Δ*Tg*CDPK3 parasites are defective in calcium-stimulated microneme secretion. A) Live fluorescent time lapse microscopy of wild-type and Δ*Tg*CDPK3 parasites, transiently expressing an ectopic DsRed protein which is targeted to the PVM, and treated with cytD to disrupt motility. Wild-type parasites permeabilize the PVM coincident with the timing of normal egress following addition of A23187, as seen by diffusion of DsRed from the PVM through the host cell. Δ*Tg*CDPK3 parasites cannot permeabilize the PVM, and DsRed remains within the PVM. Calcium ionophore is added at 30 sec time point. B) Following stimulation with A23187, wild-type parasites display microneme proteins *Tg*SUB1 and *Tg*MIC11 at the apical tip, indicative of microneme secretion, as seen by co-staining with the surface marker *Tg*SAG1. *Tg*GRA1 diffuses from the PVM through the host cell coincident with microneme secretion by wild-type parasites, confirming DsRed permeabilization results. Ionophore-treatment of Δ*Tg*CDPK3 parasites stimulates no such microneme protein secretion, and *Tg*GRA1 remains within the PVM. C) Stimulating calcium signaling in extracellular Δ*Tg*CDPK3 parasites by treatment with either 1% EtOH or A23187 shows no defect in secretion of a range of microneme proteins in supernatant samples, as compared to wild-type parasites. “*” denotes weak banding of unprocessed *Tg*MIC2 in Δ*Tg*CDPK3 supernatant samples, indicating a small level of inadvertent parasite lysis, but not sufficient to explain levels of microneme proteins seen in Δ*Tg*CDPK3 supernatant.

To assay specifically for PVM permeabilization, Δ*Tg*CDPK3 and wild-type parasites were transfected with a vector containing an ectopic DsRed protein fused to a signal peptide that permits export into the parasitophorous vacuole space. To uncouple actin-dependent gliding motility from secretion of factors controlling PVM permeabilization, parasites were treated with the actin filament disruptor cytochalasin D (cytD) [11]. As visualized by dispersion of DsRed into the host cell, wild-type parasites permeabilized the PVM ∼1.30 min after addition of A23187, coincident with the timing of normal egress (Fig. 4A, Video S4). Δ*Tg*CDPK3 mutants, however, were unable to permeabilize the PVM after ionophore stimulation, as DsRed remained trapped in the PVM with parasites (Fig. 4A, Video S5).

Given that *Tg*PLP1, the only known component required for PVM permeabilization, is located in the micronemes, we questioned whether the inability of Δ*Tg*CDPK3 parasites to activate egress was a result of a more general defect in microneme secretion. To investigate this, parasites were grown for 24 hr, again actin-based motility disrupted using cytD, and microneme release stimulated with A23187. Parasites were fixed for IFA, and host cell plasma membrane and PVM (but not parasite membrane) selectively permeabilized using 0.005% w/v saponin. This technique allows visualization of microneme proteins specifically if released onto the parasite surface [15]. We confirmed that this concentration of saponin did not permeabilize parasites by probing for cytosolic proteins (data not shown).

Using this technique, the microneme proteins Subtilisin 1 (*Tg*SUB1) and *Tg*MIC11 were observed secreted onto the surface of wild-type parasites (marked by SAG1) following ionophore stimulation, but not on DMSO-treated controls (Fig. 4B). We also stained against the dense granule protein *Tg*GRA1, which is released from the PV into the host cell upon PVM permeabilization in a *Tg*PLP1-dependent manner, similar to what is observed for PV-targeted DsRed by live microscopy [15], [49]. While wild-type parasites stimulated with A23187 showed diffuse *Tg*GRA1 staining through the host cell, indicative of PVM permeabilization, DMSO treated controls only showed *Tg*GRA1 staining within the PVM. However, Δ*Tg*CDPK3 parasites showed no detectable secretion of microneme proteins following ionophore stimulation, and no release of *Tg*GRA1 from the PVM. We could not observe a defect in microneme formation in Δ*Tg*CDPK3, suggesting it is specifically the release of microneme contents that is defective in transgenic parasites (Fig S1).

Given that we saw no block in *T. gondii* extracellular motility and invasion we were interested to see whether microneme secretion is also blocked in extracellular Δ*Tg*CDPK3 parasites. Here, stimulation of calcium signaling activates release of microneme proteins into the supernatant [50]. We assayed a range of microneme markers by western blot: microneme proteins *Tg*MIC2 [51], *Tg*MIC4 [52], *Tg*MIC5 [53], *Tg*MIC11 [54], *Tg*PLP1 [15], and *Tg*SUB1 [55]. We observed no discernible difference in levels of secreted proteins between Δ*Tg*CDPK3 and wild-type parasites after stimulation of calcium signaling with either 8 µM A23187 or 1% ethanol (Fig. 4C). The presence of only weak bands of the unprocessed form of *Tg*MIC2 in Δ*Tg*CDPK3 supernatant (Fig. 4C - “*”) indicates that microneme proteins present in the supernatant were largely due to genuine secretion, and not inadvertent lysis of parasites.

These results suggest that *Tg*CDPK3 regulates microneme secretion specifically during activation of egress from host cells, but its necessity is overcome when parasites are extracellular. This agrees with above results wherein Δ*Tg*CDPK3 mutants have no apparent defect in motility or invasion, but only in egress. This suggests a previously unappreciated complexity in apicomplexan gliding motility signaling, demonstrating a requirement for specific regulatory pathways during specific stages of parasite dissemination and host infection. It seems likely that the relative importance of these pathways for gliding motility is dictated by the parasite's local environment (i.e. whether it is exposed to intracellular or extracellular conditions).

### High potassium concentration within the host cell likely determines the requirement for *Tg*CDPK3 in calcium-dependent host cell egress

**Figure 5:**
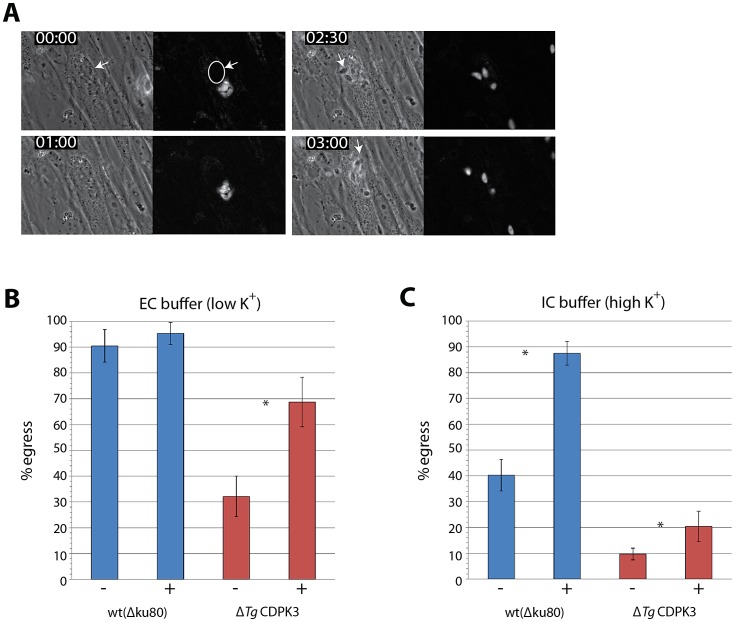
*Tg*CDPK3 kinase activity is required for activation of gliding motility in high potassium environments. A) Live time lapse microscopy of host cells co-infected with YFP-expressing wild-type and Δ*Tg*CDPK3 parasites. Δ*Tg*CDPK3 parasites are able to activate gliding motility and egress shortly after wild-type parasites escape the host cell. White arrows indicate egressing Δ*Tg*CDPK3 parasites. B) Selective saponin permeabilization of PVM and host cell membrane induces wild-type egress if treated in a low potassium (“extracellular” – EC) buffer, and is not significantly enhanced by stimulated with calcium ionophore. Δ*Tg*CDPK3 parasites show significantly lower levels of egress then wild-type whether ionophore-stimulated or not, but egress is enhanced with A23187 stimulation. C) Wild-type parasites are inhibited in permeabilization-induced egress in a high potassium (“intracellular” – IC) buffer, relative to EC buffer treatment, but this can be overcome by stimulation of calcium signaling by A23187. Δ*Tg*CDPK3 parasites are severely inhibited in egress under both conditions, and are not able to overcome inhibition following ionophore stimulation. “−” indicates A23187-negative (DMSO-treated controls) , “+” indicates A23187-treated samples. “*” indicates significant difference between DMSO- and A23187-treated samples (*P*<0.05, two-tailed Student's t-test). Error bars = ± s.d.

We next investigated the conditions under which *T. gondii* relies on *Tg*CDPK3 activity. Recently, Kafsack et al. described a knockout of *Tg*PLP1 with an egress phenotype related to an inability to permeabilize the PVM, though motility within the constraints of the PVM was normal [15]. This phenotype could be complemented with co-infection of the host cell by a wild-type strain: wild-type secretion of *Tg*PLP1 and subsequent egress disrupted both wild-type and knockout PVMs, thereby allowing ΔPLP1 egress. We were interested whether Δ*Tg*CDPK3 parasites would similarly be complemented in their egress defect by co-infection with wild-type parasites. Host cells were co-infected with Δ*Tg*CDPK3 mutants and RH parasites expressing YFP (cofilin-YFP), which are not defective in motility, invasion or egress (data not shown). Egress was then observed live using time-lapse imaging. Doing this, YFP-expressing wild-type parasites were observed to activate host cell egress 1–1.30 min after addition of A23187, and Δ*Tg*CDPK3 parasites activated gliding motility following this within 30 sec (Fig. 5A, Video S6). This suggests that extrinsic rupture of the host cell can allow normal activation of egress for Δ*Tg*CDPK3 mutants.

We therefore wondered whether rescue of the Δ*Tg*CDPK3 egress defect, by co-infection with wild-type parasites, was due to a change in the parasite ionic environment caused by host cell rupture by cofilin-YFP parasites. It has previously been demonstrated that the high potassium concentrations within the host cell, as opposed to relatively low concentrations in the extracellular medium, serves as an inhibitory signal preventing premature egress [23]. Moudy *et al.* hypothesized that, in culture, parasite-infected host cells will begin to break down as parasites replicate beyond their physical constraints. This would cause an efflux of potassium which parasites respond to by upregulating calcium signaling pathways, activating gliding motility and escaping the dying host cell. It has been demonstrated that other mutant strains which exhibit defects in A23187-induced egress can still activate gliding motility and egress normally if the host cell and PVM are selectively permeabilized with saponin, exposing parasites to the “low” potassium extracellular environment [56]. We therefore hypothesized that Δ*Tg*CDPK3 parasites are specifically defective in gliding motility and microneme secretion in a high potassium environment, but regulate calcium signaling normally in low potassium conditions.

To investigate this, infected HFFs were permeabilized with 0.005% w/v saponin diluted in high potassium “Intracellular” (IC) buffer, at 4°C. As previously described, this treatment selectively permeabilizes the host cell membrane and PVM without affecting the parasite membrane, therefore allowing control of the ionic environment that *T. gondii* is exposed to [23]. After permeabilization infected host cells were then switched either to a low (“Extracellular” – EC) or high (IC buffer) concentration potassium buffer to monitor the importance of this ion in the requirement of *Tg*CDPK3 for egress.

First assaying host cell egress by exposure to low potassium buffer, we observed that wild-type parasites efficiently activated egress and that stimulation with ionophore did not significantly amplify this response (90% ± s.d. 6.3 without ionophore, 95% ± s.d. 4.3 with) (Fig. 5B), as previously described [23]. Under the same conditions we saw that Δ*Tg*CDPK3 parasites did upregulate egress but at a much lower level, only achieving 32% (± s.d. 7.8) egress without ionophore treatment. Egress of Δ*Tg*CDPK3 could, however, be increased 2.1 fold to 69% (± s.d. 9.6) by the addition of ionophore. Interestingly, egress capacity of Δ*Tg*CDPK3 parasites did not reach the level of wild-type parasites, suggesting that even when potassium levels drop (and permeabilization of the PVM and host membrane is removed as a barrier) *Tg*CDPK3 is still required to upregulate a response to an external trigger for calcium-dependent egress. This suggested *Tg*CDPK3's activity may in fact amplify the response to a calcium-induced egress stimulus, rather than being a binary switch to turn on gliding motility.

We next tested the ability of wild-type and Δ*Tg*CDPK3 parasites to egress in a high potassium environment. Doing so we noticed that wild-type parasites were inhibited to 40% (± s.d. 6.1) egress by exposure to high potassium IC buffer, but that this inhibition could be overcome by the addition of A23187, amplifying egress 2.2 fold to the maximal amount seen in EC buffer (87% ± s.d. 4.6) (Fig. 5C). In line with our results in EC buffer we also saw that Δ*Tg*CDPK3 parasites, without addition of ionophore, could not egress as efficiently as wild-type, showing only minimal levels of egress (9.7% ± s.d. 2.3). Importantly however we saw only low levels of egress following A23187 stimulation, with parasites increasing egress by ×2.1 but remaining severely inhibited (20% ± s.d. 5.9). This supported the idea that, in a high potassium environment, *T. gondii* is much more reliant on *Tg*CDPK3 to amplify a calcium signal and activate gliding motility. Furthermore, in these experiments we considered an egressed vacuole to be one that showed one or more parasites that had moved completely free from the PVM. With this in mind we noticed that egress of wild-type parasites typically resulted in exit and dissemination by all parasites from the central vacuole, while in IC buffer Δ*Tg*CDPK3 vacuoles that were counted often showed egress of only one or two parasites (Figure S2). This supported the idea that *Tg*CDPK3 could play a role in the amplification of response to an egress stimulus, and that without its activity parasites are dysregulated in their response.

### Dual acylation is required for *Tg*CDPK3 function

**Figure 6:**
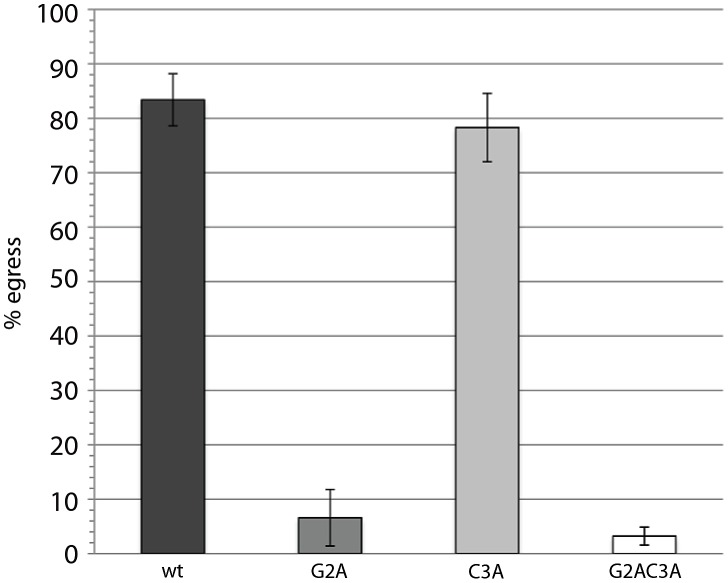
Activity of *Tg*CDPK3 requires putative acylated residues in the consensus N-terminal motif. Δ*Tg*CDPK3 parasites transfected with the wild-type (*Tg*CDPK3_wt_) or palmitoylation mutant (*Tg*CDPK3_C3A_) ectopic copies of *Tg*CDPK3 show wild-type levels of egress, following stimulation with A23187, indicating successful complementation. Complementation with myristoylation (*Tg*CDPK3_G2A_) or double mutants (*Tg*CDPK3_G2AC3A_) is not successful. Error bars = ± s.d.

We were interested in the importance of *Tg*CDPK3's N-terminal acylation sequence for its activity. To this end, Δ*Tg*CDPK3 parasites were transfected with the above-described full-length *Tg*CDPK3 cDNA N-terminal mutant constructs. This created the double mutant Δ*Tg*CDPK3 lines *Tg*CDPK3_wt_/Δ*Tg*CDPK3, *Tg*CDPK3_G2A_/Δ*Tg*CDPK3, *Tg*CDPK3_C3A_/Δ*Tg*CDPK3 and *Tg*CDPK3_G2AC3A_/Δ*Tg*CDPK3. Ionophore-induced egress was then stimulated in late stage (∼30 hr growth) double mutant parasite vacuoles with 8 µM A23187 for 3 min. By doing this, we could show that *Tg*CPK3_wt_/Δ*Tg*CDPK3 parasites were efficiently complemented, with egress levels reaching those typically seen in wild-type parasites (Fig. 6). However, *Tg*CDPK3_G2A_/Δ*Tg*CDPK3 and *Tg*CDPK3_G2AC3A_/Δ*Tg*CDPK3 mutants could not complement the calcium-stimulated egress defect. Interestingly, *Tg*CDPK3_C3A_/Δ*Tg*CDPK3 parasites also showed wild-type levels of egress, suggesting that ablation of palmitoylation does not in fact absolutely disrupt membrane localization of *Tg*CDPK3, as seen in Fig. 1B. Together, these data demonstrate that *Tg*CDPK3's localization to the plasma membrane is vital for its activity.

## Discussion

In this paper we have presented evidence that *Tg*CDPK3, a plant-like calcium dependent kinase of *T. gondii*, specifically regulates calcium-dependent activation of gliding motility during egress from host cells. While knockout of *Tg*CDPK3 left intracellular parasites unable to respond to calcium stimuli to upregulate microneme secretion, permeabilize the PVM or activate motility during egress, extracellular parasites showed no such difficulties in regulating these processes. This suggests that *Tg*CDPK3 is involved in the calcium transduction cascade activated during egress, its activity being required upstream of multiple processes. Specifically, we demonstrate that it is the ionic environment of the host cell, wherein parasites are usually switched to an inert, gliding motility “off” state (which must be overcome by upregulating calcium signaling cascades to activate egress), which determines requirement for *Tg*CDPK3 activity. Most likely it is the potassium concentration of the parasite's environment, which dictates this requirement, as the ability to activate gliding motility has been shown previously to depend on this [23]. This work therefore provides some of the first insights into the means by which *T. gondii*, and by extension other motile and invasive forms of apicomplexan species, switch from an immotile to active motile state.

*Tg*CDPK3's activity appears to require localization to the parasite plasma membrane. We predict that this localization is achieved by acylation of a consensus N-terminal motif, which is common to many plant CDPKs [24]. Although we have not definitively demonstrated acylation of this motif in *Tg*CDPK3, if it were dual acylated we would predict that the Gly2 residue is myristoylated co-translationally, which would then serve as a recognition site for reversible Cys3-palmitoylation, allowing stable association with membranes [43]. This would have two implications: myristoylation of Gly2 serving as a recognition site for Cys3-palmitoylation would mean that a G2A mutation would have the same effect on localization, and therefore activity, as a double Gly2Cys3 mutant; and ablation of Cys3 would still permit myristoylation of Gly2, but only unstable association with the plasma membrane would be possible. These predictions agree with our observed results. Neither the *Tg*CDPK3_G2A_ nor *Tg*CDPK3_G2AC3A_ variants were able to complement the Δ*Tg*CDPK3 egress phenotype, most likely because disruption of these residues completely ablated *Tg*CDPK3's plasma membrane affinity. This suggests that these mutations are in fact equivalent and N-terminal acylation is no longer possible. On the other hand, ability of *Tg*CDPK3_C3A_ to complement egress indicates that a small proportion of the *Tg*CDPK3 population can transiently interact with substrates at the plasma membrane, likely through Gly2-myristoylation, despite the majority of the kinase localizing to the cytosol. Localization of *Tg*CDPK3_NC3A_ appears to confirm this hypothesis, as a small concentration is observed at the parasite periphery. These data represent the first description of the importance of subcellular localization to the activity of an apicomplexan CDPK, or of any kinase in this phylum that we are aware of. The importance of this N-terminal motif to kinase activity furthermore suggests that *Tg*CDPK3's substrates are also embedded in the plasma membrane, although this remains to be demonstrated.

As mentioned, our data indicate that *Tg*CDPK3 is required upstream of calcium-induced microneme secretion and glideosome activation during host cell egress. This would appear to be in contradiction to the hypothesized role for its *P. falciparum* orthologue, *Pf*CDPK1, which has been shown *in vitro* to phosphorylate components of the glideosome complex, and is therefore thought to directly regulate glideosome activity through calcium-dependent phosphorylation [39]. If *Tg*CDPK3 and *Pf*CDPK1 do have related roles in *T. gondii* and *P. falciparum*, and *Tg*CDPK3 does phosphorylate the glideosome, it is certainly not in exclusion of a broader role in egress signaling. This would suggest that future work with *Pf*CDPK1, now bolstered by a range of confirmed small molecular inhibitors [57], should broaden its scope. What role *Pf*CDPK1 plays in regulating other calcium-dependent processes, and whether the local ionic environment or lytic lifecycle stage of parasites influences the requirement for *Pf*CDPK1, would now seem to be relevant questions. However, *Tg*CDPK3 appears to have a role that is more similar to the phylogenetically unrelated *Pf*CDPK5, which also controls egress but not host cell invasion [34]. The implications of this are unclear, but could suggest that unrelated regulatory proteins between *T. gondii* and *P. falciparum* have convergently evolved between the species to occupy similar functional niches.

Another reason to believe that *Tg*CDPK3 does not play a direct role in calcium-dependent phosphorylation of the glideosome is that most proteins described in *T. gondii* to have a role in activating gliding motility are required through all three forms: egress, motility, and host cell invasion [32], [58], [59]. Furthermore, a defect in glideosome activity would not be expected to affect microneme secretion. The Δ*Tg*CDPK3 phenotype therefore suggests *Tg*CDPK3 acts to modulate the signal transduction cascades that, by responding dynamically to the local environment, activate these processes in gliding motility. This of course will need to be proven, but we feel that current methodologies established in our lab will be insufficient to specifically answer the question of whether *Tg*CDPK3 is directly regulating the motor or rather is acting upstream [37].

Although the molecular mechanism of *Tg*CDPK3's role remains to be elucidated, results from the saponin-induced egress assay have allowed us to formulate a hypothesis. We observed that, during ionophore stimulation in high potassium “intracellular” buffer, Δ*Tg*CDPK3 parasites were inhibited in activation of egress, compared to the level of egress seen in wild-type parasites. This inhibition could be overcome if Δ*Tg*CDPK3 mutants were stimulated in low potassium “extracellular” buffer, though egress again did not reach that observed for wild-type parasites. We propose that multiple pathways are involved in the calcium-dependent activation of gliding motility during egress in *T. gondii*, and that *Tg*CDPK3's role is to rapidly amplify the strength of these signaling pathways to allow quick escape from a host cell.

In this scenario, treatment with calcium ionophore or a drop in the local potassium concentration both act to induce release of calcium from intracellular stores. This initial stage of calcium signaling results in a low cytosolic calcium concentration, sufficient in itself to stimulate only more sensitive calcium-induced processes, such as conoid extrusion. We hypothesize that this weak calcium flux is detected by *Tg*CDPK3, which then acts to amplify the signal by calcium-dependent phosphorylation of one or more components of the transduction cascade. Substrates may include plasma membrane-embedded ion channels, for example the sodium/hydrogen exchangers (*Tg*NHE1, *Tg*NHE2, *Tg*NHE3), which are known to be important regulators of parasite cytosolic calcium concentration [60], [61]. Rapid upregulation of calcium transduction cascades would then allow activation of microneme secretion, gliding motility, and escape from the host cell. In doing this, parasites would switch from an inert, calcium signaling and gliding motility “off” state, to an active, “on” state. If *Tg*CDPK3 is only required in amplification of signaling to achieve this state switch, it would explain why we observe no defect in Δ*Tg*CDPK3 parasites during extracellular microneme secretion, motility or invasion. They have likely already undergone this switch once in a low potassium environment, albeit the initial transition may have occurred less rapidly.

In this model, switching to EC buffer allows the activation of those signaling mechanisms utilized in gliding motility when parasites are extracellular, partially overcoming the dependency of parasites on *Tg*CDPK3 for gliding motility and explaining why we see an upregulation of egress in EC compared to IC buffer without ionophore treatment. However, the full activity of *Tg*CDPK3 is required for a rapid response to the calcium flux induced by a drop in potassium concentration, which is why egress levels are nevertheless lower than wild-type in the time scale that we measured. Interestingly, Black *et al.* have previously described a *T. gondii* mutant with an identical phenotype to Δ*Tg*CDPK3 [56]. We now know that this mutant is in fact deficient in activity of *Tg*CDPK3 (see co-submitted paper, Garrison *et al.*). Black *et al.* demonstrated that this mutant exhibited wild-type levels of egress following stimulation by permeabilization with saponin in low potassium buffer (rather than permeabilization in IC buffer followed by a transfer to EC buffer, as done by us). This may support the role for *Tg*CDPK3 in a rapid response to changes in potassium concentration, as saponin permeabilization takes 20 min of pre-incubation. Permeabilization in low potassium buffer therefore presumably exposes parasites to “extracellular” conditions for a far longer time than the 3 min used in our experiments in switching buffers for stimulation. This long incubation in low potassium conditions could allow parasites to slowly adjust, bringing gliding motility mechanisms into play in time for when egress is stimulated. This may also explain why we note a relatively high level of Δ*Tg*CDPK3 egress following DTT stimulation. DTT stimulation takes 15 min, and therefore could give a chance Δ*Tg*CDPK3 to slowly respond to the stimulus compared to the brief 3 min allowed for A23187 stimulation.

Finally, that knockout of *Tg*CDPK3 affects the ability of parasites to rapidly amplify an initially small calcium signaling response during egress could be supported by the observation that Δ*Tg*CDPK3 and wild-type parasites respond to calcium ionophore stimulation with the same fold-change in egress rate in IC buffer (roughly doubling the level of egress), although the absolute levels of egress are considerably different. Here it seems that the same initial level of calcium release can be stimulated by A23187 addition, but only wild-type parasites efficiently amplify this signal.

This work presents some of the first insights into the intricacies of calcium signaling during gliding motility in the Apicomplexa. The question now stands whether there are distinct regulators of “extracellular” calcium signal transduction cascades, and if these regulators are conversely dispensable for escape from a high potassium environment. Another obvious question is why motility in a high potassium environment is important to parasites, and why its rapid activation might be vital. The possibility that attack by the immune system may be an extrinsic inducer of calcium signaling and egress while parasites are replicating within host cells [62] could point to the biological role of *Tg*CDPK3 in *T. gondii*. Clearly, understanding the mechanism of *Tg*CDPK3's activity will be greatly enhanced by the identification of this kinase's direct substrates, which will be a focus of future work.

## Materials and Methods

### Ethics statement

Antibodies were raised in rabbits under the guidelines of the National Health and Medical Research Committee and the PHS Policy on Humane Care and Use of Laboratory Animals. Details of our procedures were approved by the WEHI animal Welfare Committee, approval number 2011.009r0

### Host cell and parasite cultures

*T. gondii* tachyzoites of RH Δ*hxgprt* (ΔHx) [63] or RH Δ*ku80* (ΔKu80) [45] strains, and all derived lines, were maintained in human foreskin fibroblasts (HFFs) in Dulbecco's Modified Eagle medium (DME) supplemented with 1% foetal calf serum (FCS) (Invitrogen), 1% v/v Glutamax (Invitrogen). Before inoculation HFFs in culture were maintained in DME supplemented with 10% cosmic calf serum (Thermo Scientific).

Transfection of *T. gondii* was performed by resuspending 1×10^7^ parasites in cytomix [64] and 10–50 µl DNA to a final volume of 400 µl. 15 µg of linearized DNA was used for tagging an endogenous genomic locus, 50 µg for introduction of ectopic plasmids. Electroporation conditions were 1.5 kV, 25 µF, 50 Ω. Parasites were transferred immediately after transfection to HFFs in complete medium. Recombinant parasites were selected by addition of either mycophenolic acid (20 µg/mL) and xanthine (50 µg/mL), or chloramphenicol (20 µM) to media as appropriate.

### Buffer composition

Extracellular (EC) and intracellular (IC) buffers were composed as previously described [23].

### α*Tg*CDPK3 antibody production

A peptide corresponding to a unique stretch of 14 amino acids in the N-terminus of the predicted *Tg*CDPK3 protein sequence was synthesized by Mimotopes (Melbourne, Australia). The resulting peptide 42- N- DSGKGTGSPDTKRD -C -56 (where numbers indicate start and end residue numbers of *Tg*CDPK3) was conjugated to keyhole limpet hemocyanin, (KLH) and used to immunize New Zealand white rabbits.

### DNA cloning

All amplification of DNA for creation of constructs was performed using PrimeSTAR proofreading polymerase (Takara), according to the manufacturer's instruction. Total RNA was extracted from *T. gondii* using an RNA extraction kit (Qiagen), and cDNA amplification performed using the Superscript first strand synthesis kit (Invitrogen), according to the manufacturer's instructions. All restriction enzymes were received from New England Biolabs, and used according to the manufacturer's instructions.

To create CDPK3-3HA, the C-terminal region of *tgcdpk3* (*toxodb.org* gene ID: TGME49_105860) was amplified from total parasite gDNA using primers 1 and 2 (Table S1). The resulting PCR product was cloned into pLIC-HA3-Hx vector [65], using the ligation independent cloning method [45]. This vector was linearized with *Sph*I prior to transfection.

The complementation constructs CDPK3_wt_-3HA-pHTU, CDPK3_G2A_-3HA-pHTU, CDPK3_C3A_-3HA-pHTU and CDPK3_G2AC3A_-pHTU were created by amplification from *T. gondii* cDNA using primers 3 (wt), 4 (G2A), 5 (C3A) or 6 (G2AC3A) together with primer 7 (Table S1). This puts the complementing *Tg*CDPK3 variants under control of 2760 bps of the tubulin upstream region. In order to stably integrate constructs at a known locus, plasmids contained a *uprt* flank. This vector also contained the HXGPRT selectable marker, 3×HA tags, while the *Tg*CDPK3 variant's expression was driven by the tubulin promoter – here named p3HA-HTU. The p3HA-HTU vector was constructed by cloning in the 3′ region of *uprt*, (*toxodb.org* gene ID: TGME49_112480) amplified from *T. gondii* gDNA by primers 8 and 9 (Table S1) and cloned in the *Kpn*I site of p3HA-HT. *Tg*CDPK3 N-terminal variant PCR fragments were cloned into *Nhe*I and *AflI*I sites of the p3HA-HTU vector, and the resulting vector linearized with *Mfe*I prior to transfection.

Plasmids containing just the N-terminal 15 amino acids of *Tg*CDPK3 fused to mOrange and HA tags were made by annealing complementary oligo pairs together followed by ligation into *Avr*II/*Bgl*II sites of pCTO3H (CAT-Tubulin 5′– mOrange-3HA). N-terminal oligo constructs *Tg*CDPK3_Nwt_-mOrange-3HA-pCT, *Tg*CDPK3_NG2A_-mOrange-3HA-pCT, *Tg*CDPK3_NC3A_-mOrange-3HA-pCT, *Tg*CDPK3_NG2AC3A_-mOrange-3HA-pCT were created by annealing complementary oligo pairs: 10 and 11; 12 and 13; 14 and 15; 16 and 17, respectively (Table S1), followed by ligation into pCTO3H.

ΔCDPK3-TOXOY72 was created by cosmid recombineering, as described previously [44]. Briefly, *toxodb.org* genome browser was used to identify end-sequenced cosmids overlapping the *tgcdpk3* gDNA sequence. Cosmid TOXOY72 was kindly provided by Prof. Boris Striepen, and transformed into EL250 *E. coli* [66]. Primers 18 and 19 were designed to amplify a chloramphenicol + gentamycin resistance cassette from vector pH 3CG, while also including 50 bp sequences homologous to regions flanking either side of the 3′ half of *tgcdpk3*. The resulting flanked-resistance cassette PCR fragments were transformed into electrocompetent EL250-TOXOY72 *E. coli*. EL250 λ-prophage recombination enzymes were induced by heatshock, causing knockout of *tgcdpk3*'s 3′half from TOXOY72 via homologous recombination with the flanked-resistance cassette. Successful recombinants were selected with gentamycin (10 µg/ml). The resulting ΔCDPK3-TOXOY72 vector was linearized with *Not*I prior to transfection into ΔKu80 parasites, and transfectants selected with chloramphenicol. Clonal parasite lines were screened for knockout by PCR with primers 1 and 2, and with 3 and 4 as a positive control (Table S1, “*”). Knockout was confirmed by western blot, using α*Tg*CDPK3.

### Southern blotting

Knockout of *Tg*CDPK3 in candidates identified by PCR were confirmed by Southern blot. DNA probe was amplified from the 5′ gDNA region of *Tg*CDPK3 using primers 20 and 21 (Table S1) and the PCR DIG Probe Synthesis Kit (Roche). ΔKu80 and Δ*Tg*CDPK3-candidate DNA was digested using either *Kpn*I (control) or *Mlu*I/*Sac*II (New England Biolabs), and fragments detected using anti-Digoxigenin and CSPD reagent (Roche) according to manufacturer's instructions.

### Immunofluorescence assay (IFA)

Antibodies and relevant concentrations (for IFA and western blot) are listed in Table S2.

Cells were fixed in 4% paraformaldehyde (PFA) in PBS (Sigma-Aldrich) for 10 min. For the detection of intracellular antigens, samples were then permeabilized with 0.1% Triton-X100 in PBS (BioRad) and then were blocked with 3%w/v BSA (Sigma-Aldrich) in PBS for 1 hr and probed with indicated primary antibodies, diluted in blocking solution, for 1 hr. After 4×5 min washes in PBS, cells were probed with Alexa conjugated fluorescent secondary antibodies (Invitrogen). After washing, slides were mounted onto microscope slides with Vectashield (Vector Labs). Images were taken with a Zeiss inverted microscope and recorded with an AxioCam MRm CCD (pixel resolution 1388×1040) using Axiovision software (Zeiss).

### *In vitro* growth competition assay

Freshly lysed Δ*Tg*CDPK3 and parental (ΔKu80) parasites were collected from culture and mixed 1∶1. ∼2.5×10^5^ 1∶1 mixed parasites were added in triplicate to culture on HFFs. ΔKu80/Δ*Tg*CDPK3 cultures were kept in continuous culture and, coincident with passage to fresh host cells when the culture fully lysed the HFF monolayer, 1×10^5^ mixed parasites were added to one coverslip in a 6-well plate and allowed to grow overnight. IFA's were subsequently performed, probing with αCAT (specifically staining Δ*Tg*CDPK3 parasites) and α*Tg*GAP45 (staining all parasites). At least 100 parasites were counted per coverslip, and the subsequent percentage of ΔKu80 and Δ*Tg*CDPK3 parasites determined by (number αCAT-stained parasites)/(number α*Tg*GAP45-stained parasites).

### Invasion assay

Two-color invasion assay was performed as described previously [14], with slight modifications. Briefly, 5×10^5^ ΔKu80 or Δ*Tg*CDPK3 parasites were added to HFFs on coverslips in a 24-well plate. Parasites were allowed to invade for 10 min, 20 min, 30 min, 1 hr or 2 hrs before fixation with 3.5% PFA in PBS, and then blocked. After blocking, cells were probed with α*Tg*SAG1, then washed, permeabilized and re-blocked. Cells were then probed with α*Tg*GAP45 and IFA completed as normal with relevant secondary antibodies. Tiled 16 bit 5×5 images were taken using Zeiss Axiovision software.

Images of SAG1- and GAP45-staining were then separately processed using FIJI software (Fiji), first by subtracting background fluorescence, then enhancing contrast, and finally by stitching tiles into single-image montages. The number of invaded ΔKu80 or Δ*Tg*CDPK3 parasites was then automatically calculated using Metamorph software (Molecular Devices), and the percentage level of invasion in each condition calculated as ((number αGAP45-stained parasites)-(number αSAG1-stained parasites))/(number αGAP45-stained parasites).

### Motility assay

Live motility was assayed as described previously [47]. Briefly, needle-passed and filtered parasites were suspended in HBSSc and allowed to settle onto poly-L-lysine (Sigma-Aldrich) coated plates for 3 min. Using a Zeiss LSM 5 Live microscope (Zeiss), live image sequences were then taken over 4 min of either untreated parasites or parasites stimulated by addition of 8 µM A23187. Movies were processed using FIJI software and numbers of motile parasites counted, and their forms of motility characterized based on standards established in literature [47].

### Immunofluorescence-based induced egress assays

1×10^5^ Parasites were added to HFFs grown on coverslips in a 6-well plate and allowed to grow for ∼30 hrs. Parasites were then stimulated using either 8 µM A23187 (Sigma-Aldrich) in DMSO diluted in DME-HEPES for 3 min at 37°C, or 5 mM DTT (Sigma-Aldrich) in DMSO diluted in HBSSc containing calcium and magnesium (and supplemented further with 1 mM MgCl_2_, 1 mM CaCal_2_, 10 mM NaHCO_3_, and 20 mM HEPES pH 7.5) for 15 min, before fixation. IFA proceeded as normal, probing with α*Tg*SAG1.

For saponin treated samples, 2.5×10^4^ parasites were added to HFFs on coverslips in a 24-well plate and grown for ∼30 hrs. Cells were then washed 1× with chilled IC buffer before selectively permeabilizing host cells with 0.005% w/v saponin (Kodak) in IC buffer, 4°C, 20 min. IC buffer+saponin was then aspirated off and one of the following solutions (pre-warmed to 37°C) added to cells in triplicate: IC buffer+8 µM A23187 in DMSO+0.005% saponin; EC buffer+8 µM A23187 in DMSO+0.005% saponin; IC buffer+equivalent volume DMSO+0.005% saponin; EC buffer+equivalent volume DMSO+0.005% saponin. Parasites were incubated at 37°C, 3 min, before fixation with 3% PFA in PBS, and IFA proceeded as normal.

### Live microscopy/analysis of egress

For analysis of live egress of ΔKu80 and Δ*Tg*CDPK3, 1×10^5^ parasites were added to 35 mm Fluorodishes (World Precision Instruments) and grown for ∼30 hrs. Live image sequences were taken as above with a Zeiss LSM 5 Live microscope (Zeiss), taking one image every 3 sec. 25 sec after beginning the image sequence, parasites were stimulated with 8 µM A23187. Movies were made using FIJI software.

Analysis of PVM rupture during egress was performed as previously published [15], with minor modifications. ΔKu80 and Δ*Tg*CDPK3 parasites were transfected with pDsRed (a gift from Vern Carruthers), and immediately added to HFFs grown on 35 mm Fluorodishes. Parasites were grown for ∼30 hrs, and parasites transiently expressing DsRed imaged and stimulated with A23187 as before, excepting that images were taken once every 10 seconds.

For wild-type/Δ*Tg*CDPK3 co-infection egress, ΔKu80 parasites expressing *Tg*Cofilin-YFP (data unpublished) were mixed 1∶1 with Δ*Tg*CDPK3, and 1×10^5^ parasites added to 35 mm Fluorodishes. After ∼30 hrs growth, parasites were imaged and stimulated with A23187 as before, taking images once every 5 seconds.

### Motility assay

Live motility was assayed as described previously [47]. Briefly, needle-passed and filtered parasites were suspended in HBSSc and allowed to settle onto poly-L-lysine (Sigma-Aldrich) coated plates for 3 min. Using a Zeiss LSM 5 Live microscope (Zeiss), live image sequences were then taken over 4 min of either untreated parasites or parasites stimulated by addition of 8 µM A23187. Movies were processed using FIJI software and numbers of motile parasites counted, and their forms of motility characterized based on standards established in literature [47].

### Western blot analysis of microneme secretion

Analysis of microneme secretion by western blot was performed as previously described [50], except that samples were incubated at the indicated conditions for 15 mins, using 4×10^8^ parasites resuspended in 100 µL DME-HEPES containing either no stimulants, 1% EtOH, or 8 µM A23187. Pellet and supernatant samples were then analyzed by western blot, probing with indicated antibodies

### Immunofluorescence analysis of microneme secretion

To monitor microneme protein secretion during parasite egress, 2.5×10^4^ parasites were added to HFFs grown on coverslips in a 24 well plate. Parasites were allowed to grow for 24 hrs, before growth medium was aspirated, and replaced with 2 µM cytochalasin-D (Sigma-Aldrich) in DMSO, diluted in DME-HEPES. Actin polymerization was inhibited by incubating parasites for 4 min, room temperature, before calcium signaling was stimulated using 8 µM A23187 diluted in DME-HEPES. Parasites were incubated at 37°C, 5 min, before fixation. Host cells were then selectively permeabilized with 0.005% saponin in PBS. Cells were blocked and IFA proceeded as normal, probing with indicated microneme protein antibodies.

## Supporting Information

Figure S1 Microneme formation in Δ*Tg*CDPK3 parasites is normal. Staining pattern of intracellular (A) *Tg*AMA1 and *Tg*MIC11, or (B) *Tg*MIC2 and *Tg*SUB1, shows no difference between wild-type and Δ*Tg*CDPK3 parasites.
(TIF)

Figure S2 Types of egress following saponin permeabilization of PVM and host cells. Egress of wild-type parasites always showed multiple parasites escaping from a single loci. In IC buffer, egress of Δ*Tg*CDPK3 typically consisted of only 1–2 parasites escaping the PVM.
(TIF)

Table S1 Primers used in this study. Primers are numbered and referenced throughout the Materials and Methods section.
(DOCX)

Table S2 Antibodies used in this study. States antibody name, concentration used and its source.
(DOCX)

Video S1 Live time-lapse microscopy of wild-type parasite egress, stimulated with A23187 at 30 sec point.
(MOV)

Video S2 Live time-lapse microscopy of Δ*Tg*CDPK3 parasites, stimulated with A23187 at 30 sec point.
(MOV)

Video S3 Live time-lapse microscopy of Δ*Tg*CDPK3 parasites, showing extrusion of conoids coincident with timing of wild-type egress, stimulated with A23187 at 30 sec point.
(MOV)

Video S4 Live time-lapse microscopy of wild-type parasite PVM permeabilization, transiently expressing PVM-targeted DsRed, pre-treated with cytD and stimulated with A23187 at 30 sec point.
(MOV)

Video S5 Live time-lapse microscopy of Δ*Tg*CDPK3 parasites, transiently expressing PVM-targeted DsRed, pre-treated with cytD and stimulated with A23187 at 30 sec point.
(MOV)

Video S6 Live time-lapse microscopy of host cell co-infected with Δ*Tg*CDPK3 and YFP-expressing parasites, stimulated with A23187 at 30 sec point.
(MOV)
