# Correction: *Tg*CDPK3 Regulates Calcium-Dependent Egress of *Toxoplasma gondii* from Host Cells

**DOI:** 10.1371/annotation/3be1f9af-adb8-41cb-9c03-e218e57e025c

**Published:** 2013-01-03

**Authors:** James M. McCoy, Lachlan Whitehead, Giel G. van Dooren, Christopher J. Tonkin

This article was intended for simultaneous publication with "A Forward Genetic Screen Reveals that Calcium-dependent Protein Kinase 3 Regulates Egress in Toxoplasma" on November 29, 2012 (http://dx.plos.org/10.1371/journal.ppat.1003049). It was published on December 4, 2012 due to journal error.

