# Supplementary figures and images for: TgCDPK3 Regulates Calcium-Dependent Egress of Toxoplasma gondii from Host Cells
Source: PLoS Pathog. 2012 Dec 4;8(12):e1003066. doi: 10.1371/journal.ppat.1003066 (PMC3514314; doi:10.1371/journal.ppat.1003066)

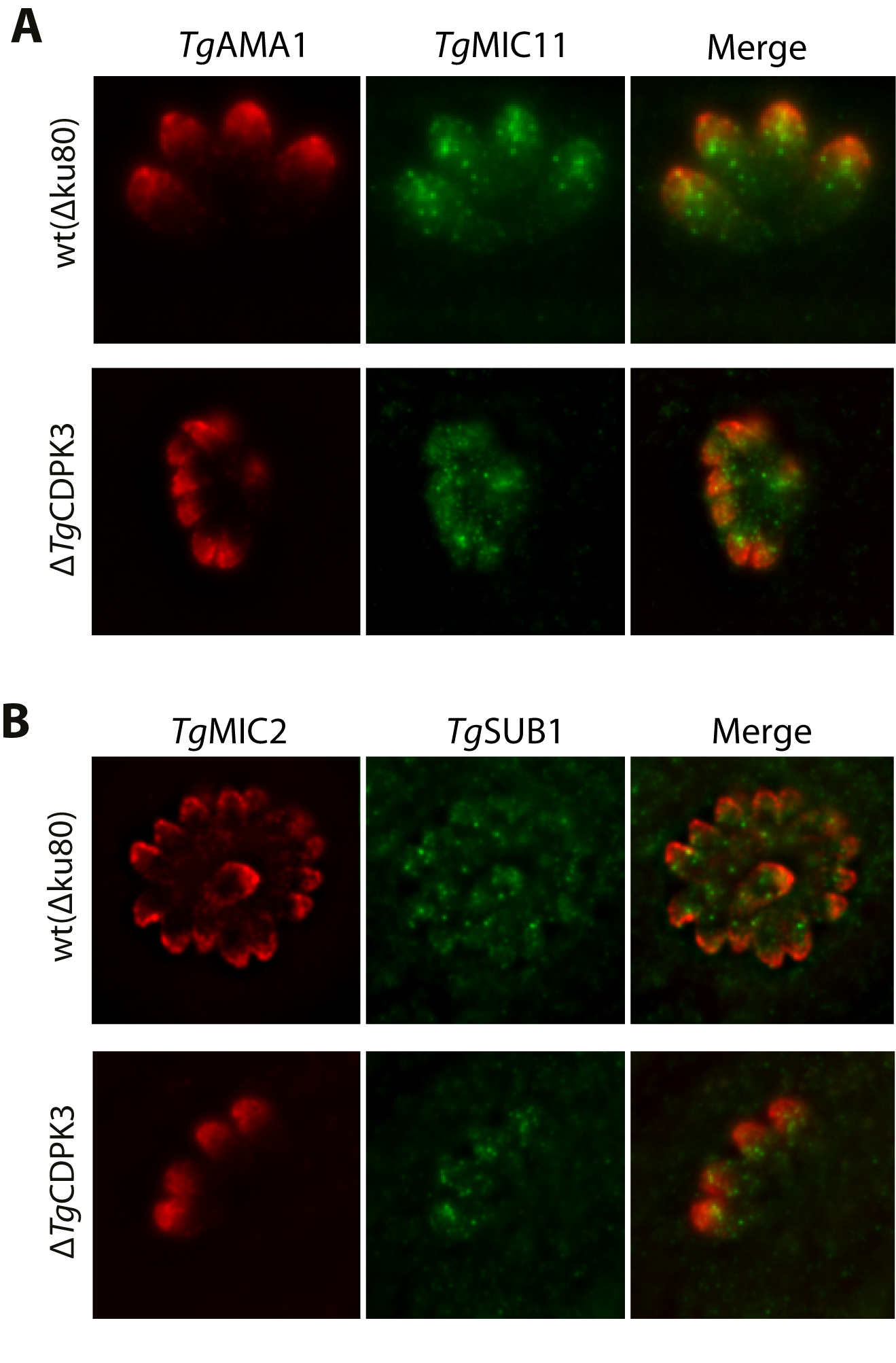

Supplement: Figure S1 — Microneme formation in ΔTgCDPK3 parasites is normal. Staining pattern of intracellular (A) TgAMA1 and TgMIC11, or (B) TgMIC2 and TgSUB1, shows no difference between wild-type and ΔTgCDPK3 parasites. (TIF) [file ppat.1003066.s001.tif]

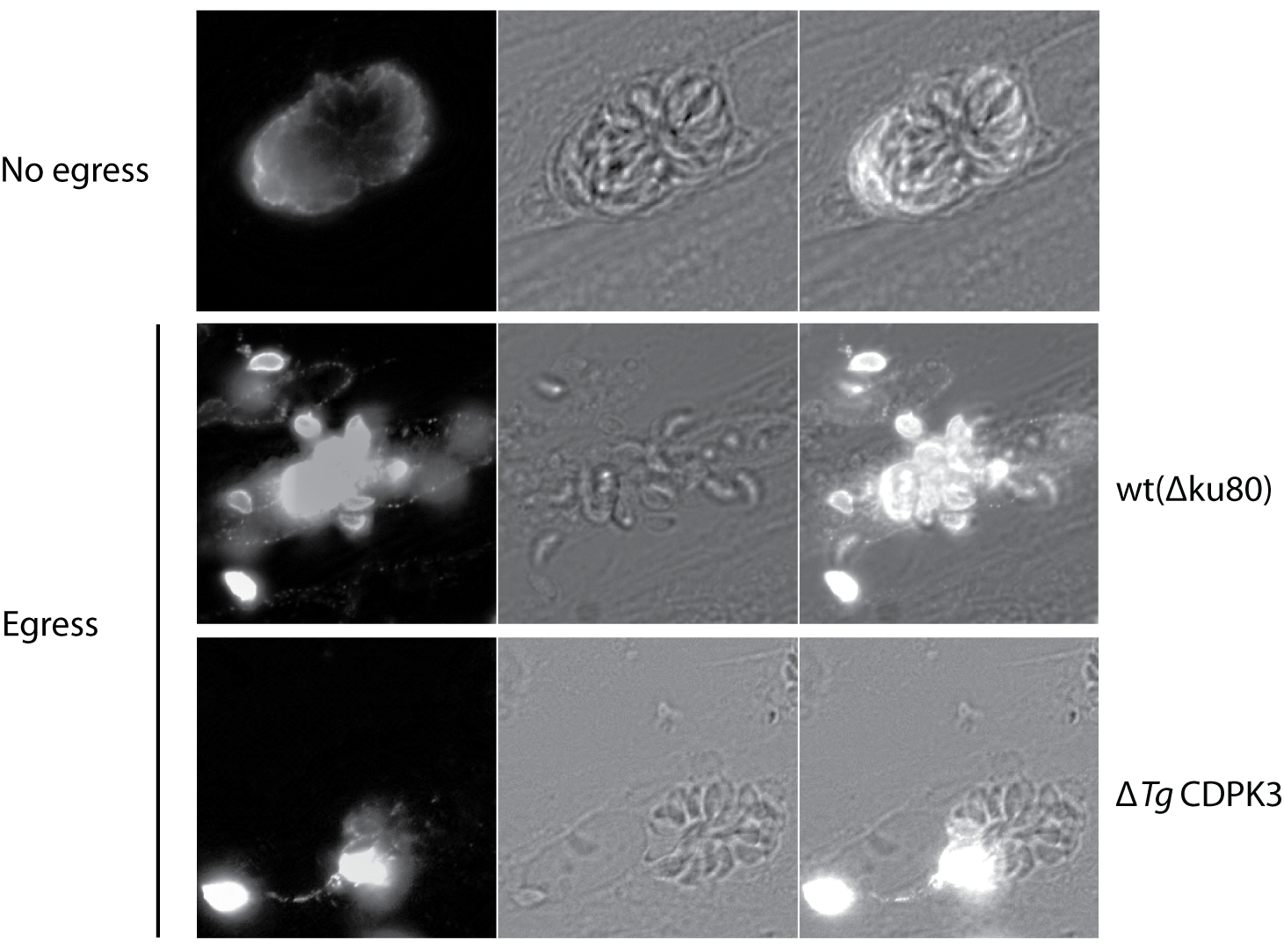

Supplement: Figure S2 — Types of egress following saponin permeabilization of PVM and host cells. Egress of wild-type parasites always showed multiple parasites escaping from a single loci. In IC buffer, egress of ΔTgCDPK3 typically consisted of only 1–2 parasites escaping the PVM. (TIF) [file ppat.1003066.s002.tif]
