# Supplementary material for: TgCDPK3 Regulates Calcium-Dependent Egress of Toxoplasma gondii from Host Cells
Source: PLoS Pathog. 2012 Dec 4;8(12):e1003066. doi: 10.1371/journal.ppat.1003066 (PMC3514314; doi:10.1371/journal.ppat.1003066)
Supplement: Table S1 — Primers used in this study. Primers are numbered and referenced throughout the Materials and Methods section. (DOCX) [file ppat.1003066.s003.docx]

| Primer ID | Sequence (5’-3’) |
| --- | --- |
| 1 | TACTTCCAATCCAATTTAGCGAGGAGTAAATTTCACGTGTCTCGAC |
| 2 | TCCTCCACTTCCAATTTTAGCGTGCTTCACTTTGACGTCGCAGATCT |
| 3 | ATGCGCTAGCATGGGGTGCGTCCACTCC |
| 3 | ATGCGCTAGCATGGCGTGCGTCCACTCC |
| 5 | ATGCGCTAGCATGGGGGCCGTCCACTCC |
| 6 | ATGCGCTAGCATGGCGGCCGTCCACTCC |
| 7 | AGTCCTTAAGGTGCTTCACTTTGACGTCGCAG |
| 8 | ATGCGGTACCGTAGAACAGACAGACGAGG |
| 9 | ATGCGGTACCGGGTAGAAATCGTACTCG |
| 10 | [Phos]-GATCTATGGGGTGCGTCCACTCCAAGAATCCCCACTCCAAGCATGCAGGCC |
| 11 | [Phos]-CTAGGGCCTGCATGCTTGGAGTGGGGATTCTTGGAGTGGACGCACCCCATA |
| 12 | [Phos]-GATCTATGGCGTGCGTCCACTCCAAGAATCCCCACTCCAAGCATGCAGGCC |
| 13 | [Phos]-CTAGGGCCTGCATGCTTGGAGTGGGGATTCTTGGAGTGGACGCACGCCATA |
| 14 | [Phos]-GATCTATGGGGGCCGTCCACTCCAAGAATCCCCACTCCAAGCATGCAGGCC |
| 15 | [Phos]-CTAGGGCCTGCATGCTTGGAGTGGGGATTCTTGGAGTGGACGGCCCCCATA |
| 16 | [Phos]-GATCTATGGCGGCCGTCCACTCCAAGAATCCCCACTCCAAGCATGCAGGCC |
| 17 | [Phos]-CTAGGGCCTGCATGCTTGGAGTGGGGATTCTTGGAGTGGACGGCCGCCATA |
| 18 | GCGGCTACCCGCCCTTCGGAGGTCAAACCGACCAGGAGATCCTCAAGAGGCCTCGACT  ACGGCTTCCATTGGCAAC |
| 19 | GTCATGTTACGTGGCGCGTACAAAACACACACAAGACGCTTCGAACGGACATACGACT  CACTATAGGGCGAATTGG |
| 20 | TTGTCTCGACTTGAGC |
| 21 | TGCATGCTTGGAGTGG |
| 1* | ACATGTGTTTTACTGCGACG |
| 2* | CTGCATGCAGGAATTCCAGTCC |
| 3* | GTAAGTCCTGTTTATTCCAGCG |
| 4* | AGCGGCTAGGTCAACATGAACC |

* PCR screening primers.
