# Supplementary material for: TgCDPK3 Regulates Calcium-Dependent Egress of Toxoplasma gondii from Host Cells
Source: PLoS Pathog. 2012 Dec 4;8(12):e1003066. doi: 10.1371/journal.ppat.1003066 (PMC3514314; doi:10.1371/journal.ppat.1003066)
Supplement: Table S2 — Antibodies used in this study. States antibody name, concentration used and its source. (DOCX) [file ppat.1003066.s004.docx]

| Antibody | Dilution | Source |
| --- | --- | --- |
| M αCAT | 1:1000 | Abcam |
| M α*Tg*GRAI | 1:2000 | Marie-France Cesbron-Delauw, IJR, France [69] |
| M αHA | 1:1000 | Roche |
| M α*Tg*MIC2 | 1:2000 (IFA), 1:5000 (western blot) | David Sibley, Univ. Wash, USA [51] |
| M α*Tg*SAG1 | 1:1000 | David Sibley, Univ. Wash, USA [68] |
| Rb α*Tg*CDPK3 | 1:500 | (See Materials and Methods) |
| Rb α*Tg*GAP45 | 1:500 | Beckers (Univ. Nth Carolina, USA) [10] |
| Rb α*Tg*MIC4 | 1:1000 | Dominique Soldati-Favre, Univ. Geneva, Switzerland [52] |
| Rb α*Tg*MIC5 | 1:1000 | Vern Carruthers, Univ. Mich, USA [53] |
| Rb α*Tg*MIC11 | 1:500 | Vern Carruthers, Univ. Mich, USA [54] |
| Rb α*Tg*PLP1 | 1:500 | Vern Carruthers, Univ. Mich, USA [15] |
| Rb α*Pf*SUB1 | 1:1000 | Mike Blackman, NIMR, UK [67] |
